# Diversity Training With Robots: Perspective-Taking Backfires, While Sterotype-Suppression Decreases Negative Attitudes Towards Robots

**DOI:** 10.3389/frobt.2022.728923

**Published:** 2022-03-09

**Authors:** Ricarda Wullenkord, Friederike Eyssel

**Affiliations:** Applied Social Psychology and Gender Research, CITEC, Bielefeld University, Bielefeld, Germany

**Keywords:** diversity training, human-robot interaction, human-robot teams, attitudes toward robots, robot attitudes, robots at the workplace, enlightenment approach

## Abstract

The present research investigated the effects of a diversity training intervention on robot-related attitudes to test whether this could help to manage the diversity inherent in hybrid human-robot teams in the work context. Previous research in the human-human context has shown that stereotypes and prejudice, i.e., negative attitudes, may impair productivity and job satisfaction in teams high in diversity (e.g., regarding age, gender, or ethnicity). Relatedly, in hybrid human-robot teams, robots likely represent an “outgroup” to their human co-workers. The latter may have stereotypes towards robots and may hold negative attitudes towards them. Both aspects might have detrimental effects on subjective and objective performance in human-robot interactions (HRI). In an experiment, we tested the effect of an economic and easy to apply diversity training intervention for use in the work context: The so-called enlightenment approach. This approach utilizes perspective-taking to reduce prejudice and discrimination in human-human contexts. We adapted this intervention to the HRI context and explored its impact on participants’ implicit and explicit robot-related attitudes. However, contrary to our predictions, taking the perspective of a robot resulted in more negative robot-related attitudes, whereas actively suppressing stereotypes about social robots and their characteristics produced positive effects on robot attitudes. Therefore, we recommend considering potential pre-existing aversions against taking the perspective of a robot when designing interventions to improve human-robot collaboration at the workplace. Instead, it might be useful to provide information about existing stereotypes and their consequences, thereby making people aware of their potential biases against social robots.

## 1 Introduction

Even though we are confronted with diversity in all sectors of our daily lives, we mostly have not considered robots as a new social group that potentially extends the notion of diversity (Ferrari and Eyssel, 2006). Endorsing stereotypes and prejudice associated with groups other than our own, however, pose psychological obstacles to positive interactions. As demonstrated in a vast amount of research from social psychology, stereotypes, and prejudice lead to various problems, e.g., an uneven distribution of resources (Abraham, 2003), and even dehumanization, the denial of full humanness (Haslam and Loughnan, 2014). Having stereotypes and being prejudiced towards a certain group encompasses negative attitudes towards the respective group (e.g., Desforges et al., 1991; Simon, 1998). Diversity plays out at the workplace as well: People from different age, gender, and cultural groups work together on a daily basis. However, pre-existing stereotypes associated with, for instance, age, gender, or ethnicity may hinder mixed teams from unfolding their full potential (e.g., Bezrukova et al., 2009; Ely et al., 2012). To illustrate, team members of one joint team may at still identify as members of various separate ingroups (e.g., Christians vs. Muslims, People of Color vs. White people etc., Dovidio et al., 2004). Stereotype threat (e.g., Walton et al., 2015) may likewise impair the performance of individuals from stereotyped groups. When people are aware that they might be evaluated in a negative light based on preexisting negative stereotypes, they tend to underperform (e.g., Inzlicht and Schmader, 2012). Above and beyond the obvious benefits of diversity management for the stereotyped group, members of the nonstereotyped majority group also benefit from strategies to manage diversity, e.g., by having a higher job satisfaction (Jansen et al., 2016). Furthermore, a company benefits from diversity management by positively impacting its stock market value (Wright et al., 1995), and the affective commitment of its employees (Ashikali and Groeneveld, 2015).

In light of the fact that in the future, robots will eventually be deployed in various domains of life and society, yet another level of complexity is added to the notion of diversity. Plausibly, hybrid human-robot teams might not only have to address preexisting stereotypes and prejudice about human team members who stem from diverse backgrounds, but such hybrid teams also need to cope with negative attitudes and stereotypes which prevail about robots. A potential solution for dealing with the latter stereotypes and prejudice may be offered by diversity training.

### 1.1 Diversity Training in the Human-Human Workplace Context

The term diversity training describes various types of interventions with the goal of “facilitating positive intergroup interactions, reducing prejudice and discrimination, and enhancing the skills, knowledge, and motivation of participants to interact with diverse others” (Bezrukova et al., 2016, p. 1228). These interventions can be applied at the workplace, where they can help raise job satisfaction, acceptance of the organization, or career progress (e.g., Hays-Thomas, 2004; Kalinoski et al., 2013). There is a vast number of approaches to diversity training that can be utilized in and outside of the workplace context when mixed or hybrid teams work together. To illustrate the concept of diversity training, we will describe types of interventions commonly utilized in the human-human context, namely information-based, guilt-based, social identity based, and empathy-based approaches.

One starting point to reduce stereotypes and prejudice by means of diversity training is to provide information about the stereotyped outgroup. This is supposed to raise awareness of existing prejudice (Pendry et al., 2007). However, as stereotypes and prejudice are often based on affect, a mere presentation of information may not be sufficient to lead to attitude change or behavioral change (Pendry et al., 2007). Another path to stereotype reduction relies on triggering guilt to make existing prejudice salient for the - usually more privileged - ingroup (Pendry et al., 2007). The experience of guilt by those high in prejudice is supposed to induce self-reflection, resulting in behavioral, and, potentially, attitudinal change (e.g., Monteith, 1993; Amodio et al., 2007). Diversity training makes use of guilt-inducing strategies, for instance, in the context of the “privilege walk” (McIntosh, 2015). The privilege walk is based on McIntosh’s work on White Privilege (1988), which is defined as a) “having greater access to power and resources than people of color” (Kendall, 2012, p. 45), and b) “the power to […] move through the world without your race defining your interactions, […] the power to remain silent in the face of racial inequity [...]. It’s knowing that you and your humanity are safe” (Collins, 2018, p.9). In this ‘privilege walk’ exercise, statements reflecting increasingly intense prejudice and discrimination are read out aloud (e.g., If a traffic cop pulls me over [...], I can be sure I have not been singled out because of my race) in an ethnically mixed group. All people to whom the respective statement applies take a step forward (e.g., Irby-Shasanmi et al., 2012). It becomes evident that the privileged group steps forward with a discernibly higher frequency, which, in turn, reveals the existing prejudice and discrimination against the outgroup. Another strategy that likewise relies on the induction of guilt is the so-called “brown eyes/blue eyes” exercise (e.g., Stewart et al., 2003). This approach is more general in nature, as it is not based on participants’ ethnicity in particular. Instead, two minimal groups are formed based on a socially irrelevant feature, that is, an individual’s eye color. In this intervention, one randomly chosen group is treated badly by another more privileged group, which presumably triggers guilt in the privileged group and should lead to the aforementioned changes in behavior and/or attitude. In their study, Stewart et al. found that participation in the exercise was connected to more positive attitudes to Asian American and Latin American individuals, and that participants recognizing themselves discriminating led to negative emotional responses, i.e., anger toward themselves.

Apart from offering information or triggering guilt, the social identity of members of a given team can also serve as a starting point for a diversity training intervention at the workplace. The “Actualizing Social and Personal Identity Resources to enhance organizational outcomes”–Model (i.e., the ASPIRe Model; Haslam et al., 2003) takes advantage of this idea. To illustrate, an intervention based on this framework would facilitate that members of a team identify as *one* ingroup (i.e., members of the organization), instead of self-categorizing as members of different ingroups (Dovidio et al., 2004). According to the ASPIRe model, participants pass through four stages. The first stage is centered around finding out which social identities the people have (AIRing). In the two following stages (Sub-Casing and Super-Casing) relevant goals for these identities are established for a) the subgroups and b) the whole organization. The final phase (ORGanizing) builds upon the outcomes of the previous two phases and adapts organizational planning and organizational goals accordingly, while the process outcomes as well as the employees’ satisfaction and commitment are supervised (see Haslam et al., 2003, p. 87). In these subsequent phases, participants are supported in assigning themselves to a joint ingroup: Being a member of the organization they work in. Consequently, the group members internalize the corresponding norms and values. However, diversity training based on this model is rather time-consuming and requires a lot of planning and effort from the respective organization.

A final potential method to reduce the negative effects of stereotypes via diversity training is perspective-taking. Perspective-taking is utilized in the enlightenment approach (Dovidio et al., 2004), which is supposed to build emotional empathy by perspective-taking to reduce prejudice and discrimination. Perspective-taking allows, for example, to focus on situational or momentary influences on the behavior of a person instead of relying on a broader or more general evaluation one might have formed based on a pre-existing stereotypes. This leads to a more personalized viewpoint when making judgments about a target (Galinsky and Moskowitz, 2000).

Even though there are reports about negative outcomes associated with diversity trainings, e.g., defensive reactions by the majority group (Kidder et al., 2004), or stronger animosity towards minorities (Sanchez and Medkik, 2004), such interventions generally prove efficient and beneficial (Kalinoski et al., 2013). Furthermore, even though diversity training is a well-tested tool for diversity management and might potentially be beneficial regardless of the identity of the team members, research till now has focused on organizational groups that consist of humans and has not considered the possibility of hybrid human-robot teams.

### 1.2 Diversity Training in the Human-Robot Workplace Context

Considering the ongoing technical development, it is highly probable that there will be mixed teams of human and robot co-workers in the future, creating a new form of diversity in the workplace. Take the automotive industry - in this context, humans have co-worked with robots for a long time (Choi et al., 2010). Apart from a high number of industrial robots that are used already, professional service robots to support workers are on the rise as well: It is estimated that till 2023, the worldwide market will grow another 31% (International Federation of Robotics, 2020). But this potential new form of diversity at the workplace does not come without pitfalls, particularly due to pre-existing stereotypes, prejudice, and negative attitudes.

Even though robots offer a vast potential to support people in the workplace by, for example, increasing productivity and flexibility (Fragapane et al., 2020), or by reducing the strain on workers in the care sector (Blake, 2019), attitudes towards robots are not as positive as one would think (e.g., Bernotat and Eyssel, 2018). Relatedly, stereotypes pose a problem for successful hybrid human-robot teams: It has been shown that stereotypes that apply within the human-human context are also readily transferred to robots (e.g., Eyssel and Hegel, 2012; Bernotat et al., 2019), biasing judgments about them. In the study by Bernotat et al. (2019), for example, the robot waist-to-hip ratio was manipulated and as hypothesized it was found that the perceived robot gender led to preferences to use the robot for stereotypically female tasks, as well as to higher evaluations of communality. Specific robot-related stereotypes, e.g., regarding a robot’s warmth and competence affect emotional responses to robots which, in turn, predict behavioral tendencies (e.g., Mieczkowski et al., 2019; Oliveira et al., 2019; see also Fiske, 2018 and Fiske et al., 2007 for research on warmth and competence perceptions in humans). Oliveira et al. (2019) manipulated robot warmth and competence in an interactive game with or against the participant and measured their emotional responses and their contact intentions. They found that, in fact, warmth and competence were related to emotional responses, specifically feelings of admiration and contempt, and that participants preferred future interactions with robots perceived as warm. This is especially important since robots are usually rated as rather competent and cold (i.e., a trait combination that is related to envious reactions, Fiske, 2018). Evaluations of someone as lacking warmth are associated with a higher perceived likelihood that the individual could behave harmfully (Fiske et al., 2007). The idea that people rate robots as cold and, consequently, as potentially more harmful than an individual or entity high in warmth relates to research suggesting that people tend to feel anxiety towards robots (e.g., Nomura and Kanda, 2003; Bartneck et al., 2005; Horstmann and Krämer, 2019). Negative attitudes and anxiety towards robots negatively influence HRI. For instance, by preventing people from interacting with robots, (Nomura et al., 2006a; Nomura et al., 2008; De Graaf et al., 2013), or by influencing whether participants react to or choose to ignore the presence of robots in an interaction (e.g., Hinz et al., 2019). Hinz et al. let people play a game with a robot in which they were supposed to prevent a balloon from exploding. They found that the more negative robot attitudes were in participants, the less they took the robot into consideration when it joined the game. Thus, such negative outcomes greatly hinder the envisioned human-robot collaboration scenarios with hybrid human-robot teams.

To reduce stereotypes and negative attitudes towards robots, we tested a diversity training approach that has been successfully used in the human-human context. To our knowledge, to date there are no studies on diversity training in the context of HRI, yet. In the present experiment, we thus used the enlightenment approach (Dovidio et al., 2004) with robot targets. The enlightenment approach is a rather simple, neither cost nor time-intensive approach that can be used in the workplace context as well as otherwise. As such it appeared ideal for a short-term lab-based study with a limited number of robots available.

### 1.3 Application of the Enlightenment-Approach in the Human-Robot Context

To test diversity training in the context of social robots, we utilized the enlightenment approach (Dovidio et al., 2004) and replicated and extended a classic experiment by Galinsky and Moskowitz (2000) which tested the influence of perspective-taking on the reduction of stereotypes and stereotype-related negative associations. In their experiment, the authors presented participants with an image of an elderly man and asked them to write a story about a day in his life. Participants were either instructed to take the perspective of the person, or to suppress their stereotypes when writing the story. The control group did not receive any further instructions. Perspective-taking reduces the accessibility of existing stereotypes, and thereby diminishes their impact (Brewer, 1996). Stereotype suppression, on the other hand, focuses the attention on existing stereotypes (Macrae et al., 1994), making them even more accessible. Galinsky and Moskowitz (2000) assessed explicit and implicit attitudes, which we adopted for our study. To illustrate, attitudes can be measured directly via self-reports, or indirectly, e.g., via reaction times (Gawronski and De Houwer, 2014). Direct vs. indirect attitude measures capture different aspects of attitudes, in that direct attitude measures capture explicit attitudes, while indirect attitude measures capture implicit attitudes (Gawronski and De Houwer, 2014). Implicit attitudes can be defined as reactions which reflect processes that are not controlled, conscious, and intentional, and work without resources required for attention (Bargh, 1994). Expressing explicit attitudes, on the other hand, requires individuals to be aware of their attitudes, being willing to report them, and willing and able to engage in the necessary introspection to report them (Gawronski and De Houwer, 2014). Implicit and explicit attitudes predict differential facets of behavior. That is, implicit attitudes are more strongly related to automatic behavior, whereas explicit attitudes are more strongly related to deliberate behavior (e.g., Friese et al., 2008). Therefore, it is beneficial to include both types of measures. Furthermore, direct attitude measures are known to potentially fall victim to biases stemming from self-presentation and/or social desirability, which in this case can be defined as the participant’s wish to leave a good impression or to solve the given task correctly (e.g., Gawronski and De Houwer, 2014). That is not as much the case for indirect attitude measures (e.g., Fazio and Olson, 2003; Petty et al., 2008). To measure implicit robot attitudes, we used an Implicit Association Test (IAT; Greenwald et al., 1998; adapted to robots: Wullenkord, 2017). This task is based on reaction times to give insights into the relative preference of one concept over another. In the present research we differentiated robots vs. humans. In addition to explicit and implicit robot attitudes, we were interested in the effect of the diversity training intervention on anthropomorphism. Anthropomorphism is defined as the attribution of human characteristics to nonhuman entities (Epley et al., 2007) and the anthropomorphizing of robots is positively related to robot attitudes (e.g., Spatola and Wudarczyk, 2020). To capture this concept, we included mind attribution (Gray et al., 2007) as a measure of anthropomorphism in the current experiment. High levels of mind attribution imply that robots are seen as more human, compared to if less mind is attributed to them. People differ in the extent to which they humanize nonhuman entities. To explore this aspect further, we measured participants’ disposition to anthropomorphize as a control variable (Waytz et al., 2010). We further included acceptance and willingness to use new technologies (Neyer et al., 2012), and robot experience, as it is likely that a higher acceptance of new technologies as well as prior robot experience influence robot attitudes and robot anxiety.

In line with Galinsky and Moskowitz (2000), we formulated the following hypotheses:

Participants would report more positive explicit and implicit negative robot attitudes, and would attribute more mind to robots if they a) took the perspective of a robot compared to participants who suppressed their stereotypes as well as compared to a control group without further instruction (H1a), and b) if they suppressed their stereotypes compared to the control group (H1b). In addition, we hypothesized that participants would report more positive explicit and implicit robot attitudes, and would attribute more mind to robots from pre-intervention (T1) to post-intervention (T2) a) if they took the perspective of the robot (H2a), and b) if they suppressed their stereotypes (H2b).

## 2 Materials and Methods

### 2.1 Design and Sample

Following the design by Galinsky and Moskowitz (2000), the study was planned as a mixed design with one between-subjects factor (perspective-taking task vs. stereotype suppression task vs. control task) and a within-subjects factor (*measurement time*: the dependent variables were assessed pre and post intervention). We aimed to collect data from *N* = 60 people, with *n* = 20 per experimental group. However, we had to pause data collection at the end of 2019 and could not resume and finalize data collection in spring of 2020 due to the Covid-19 Pandemic. Consequently, the laboratory experiment was terminated with data from *N* = 56 individuals recruited at Bielefeld University and Bonn University. One female participant had to be excluded due to too many outliers (outlier values on > 5% of variables, outliers were defined as values differing more than three standard deviations from the scale mean of the sample). Therefore, the final sample consisted of *N* = 55, 28 males, and 27 females. Participants ranged in age from 18 to 37 (*M* = 24.33, *SD* = 4.30). 52 participants were German, 48 participants were students. With regards to robot experience, participants generally had little to no robot experience (*M* = 2.76, *SD* = 1.91). 67.3% of participants rated their experience below the scale midpoint of 4, with the biggest group reporting no robot experience at all (38.2%).

### 2.2 Procedure

This laboratory experiment was conducted in a double-blind mode, meaning that neither participants nor experimenter knew whether a participant was assigned to an experimental or to the control group. The experimental procedure was approved by the Bielefeld University Ethics Board (application no. EUB-2018-198 W1). Data were collected computer-based in labs at Bielefeld University and Bonn University. Participants were recruited face-to-face and by means of flyers and advertisement on social networks like Facebook. After providing informed consent, participants read the cover story, which explained that they would work on various unrelated tasks on the topics attitudes and speech comprehension. Afterwards, they were seated in front of a laptop and filled out the pre-intervention dependent measures as well as the control variables and the demographics questionnaire. Afterwards they saw a picture of the NAO robot and were asked to write a story about its everyday life, which was the experimental manipulation. They were either asked to write the story avoiding typical stereotypes and prejudice towards robots (stereotype avoidance group, *n* = 20), by putting themselves in the robot’s position (perspective-taking group, *n* = 18), or without any further instruction (control group, *n* = 17). Afterwards, they filled out the dependent measures again for the post-intervention measurement. Finally, they were debriefed in written form and were reimbursed with course credit, if applicable.

### 2.3 Between- and Within-Subjects Factors

The operationalization and adaptation of the diversity training was based on existing research by Galinsky and Moskowitz (2000). Diversity training was varied between subjects, so that participants were randomly assigned to one of three experimental groups: A perspective-taking group vs. a stereotype suppression group vs. a control group. A within-subjects factor was introduced by measuring the dependent variables pre- and post-experimental treatment. All participants were shown a picture of a NAO robot and were asked to write a story about this robot’s everyday life. In the perspective-taking group, participants were asked to put themselves into the place of the robot and describe the everyday life from their point of view. Some participants even wrote their story in a first-person perspective. In the stereotype avoidance group, participants were asked to avoid typical stereotypes about and prejudice towards robots in their story. In the control group, participants did not receive any further instruction about what to consider when writing the story.

### 2.4 Dependent Measures

#### 2.4.1 Direct Attitude Measures

Responses on the dependent measures were provided using 7-point Likert scales, with 1 indicating low endorsement, and 7 indicating high endorsement of the measured construct.

Dependent measures were administered pre-intervention (T1) as well as post-intervention (T2). Participants indicated their attitudes towards robots on the 15-item Negative Attitudes toward Robots Scale (NARS, Nomura et al., 2004), an example item read “I would be nervous if I had to operate a robot in front of other people”. Robot anxiety was measured by the 10-item Robot Anxiety Scale (RAS, Nomura et al., 2006b), which consists of items such as “I am worried about how fast a robot would move”. Mind attribution to robots was captured by a short ten-item version of the Mind Attribution Scale (MAS, Gray et al., 2007); e.g., “To what extent are robots able to experience joy?”). Furthermore, participants answered six items on their contact intentions towards robots (Eyssel and Kuchenbrandt, 2012), an exemplary item read: “How much would you like to have a robot at home?”. In addition, they filled out eight items on their willingness to engage in future interactions with robots (Eyssel and Kuchenbrandt, 2012), using items like “Would you use a robot as a fitness coach?”. Liking of robots was measured with seven items based on Higgins et al. (2007). An example item read: “How close do you feel to robots?”.

#### 2.4.2 Indirect Attitude Measures

In addition to the measures of explicit attitudes, participants worked on a task measuring their implicit attitudes. To do so, they completed an Implicit Association Test, an indirect attitude measure (Greenwald et al., 1998) based on reaction times to assess the preference of one concept over another. The present IAT task contrasted the relative preference for humans over robots (see Wullenkord, 2017). The test consisted of seven blocks, including trial blocks in which participants were able to get used to the task, as well as the test blocks which were used to determine the implicit robot attitude. In the first test blocks we paired human silhouettes with positive words, e.g., joy or happy, and robot silhouettes with negative words, i.e., hate or angry. These were followed by test blocks which paired human pictures with negative words and robot pictures with positive words. The stimuli used in the IAT can be found in the Supplementary Material. The reaction times regarding the pairing of concepts allow to measure whether participants preferred humans or robots. After the experimental manipulation, the dependent measures were administered a second time as a post-intervention measurement (T2).

### 2.5 Covariates

To make sure that the results of the main analyses were indeed due to the experimental manipulation and not due to the unwanted impact of third variables, three covariates were included in the design of the present experiment. This was done to control for their impact on the experimental results. We administered 12 questions on acceptance of and willingness to use new technologies (TA; Neyer et al., 2012), i.e., “I am curious toward new technical developments”, followed by nine items on participants’ Individual Differences in Anthropomorphism (IDAQ, Waytz et al., 2010). The IDAQ measures participants’ tendency to anthropomorphize nature, animal world, and technology: “To what extent does the average robot have consciousness?”. Both scales were measured before and after the experimental manipulation Finally, participants indicated their prior experience with robots.

## 3 Results

We were interested in the effects of diversity training with a robot, more specifically of taking the perspective of a robot, on explicit and implicit robot attitudes, robot anxiety, and anthropomorphism. We therefore instructed participants to write about the daily life of a robot, by taking the robots perspective vs. suppressing stereotypes vs. no further instruction (control group). We predicted that participants would report more positive explicit and implicit negative robot attitudes, and would attribute more mind to robots if they a) took the perspective of a robot compared to participants who suppressed their stereotypes as well as compared to a control group without further instruction (H1a), and b) if they suppressed their stereotypes compared to the control group (H1b). In addition, we hypothesized that participants would report more positive explicit and implicit robot attitudes, and would attribute more mind to robots from pre-intervention (T1) to post-intervention (T2) a) if they took the perspective of the robot (H2a), and b) if they suppressed their stereotypes (H2b).

### 3.1 Data Preparation: IAT Scoring

To prepare the IAT data for analyses, a D score was computed using the D-algorithm (Greenwald et al., 2003). The D score reflects a relation between the reaction times in ‘consistent’ trials (i.e., trials in which human pictures were paired with positive words and robot pictures with were paired with negative words), and the reaction times in ‘inconsistent’ trials (i.e., the trials in which human pictures were paired with negative words and robot pictures were paired with positive words, respectively). If this score is significantly different from zero, this means that participants showed a preference of one group over the other. In the context of our experiment, this would imply that participants preferred humans over robots. If the score is not different from zero, in this experimental context that would mean that humans and robots are evaluated equally positive or negative, respectively.

### 3.2 Preliminary Analyses

#### 3.2.1 Internal Consistencies

To gain insights into the psychometric characteristics of the measures used in the present research, internal consistencies (Cronbach’s α) were computed and are displayed in Table 1. All reliabilities for the direct measures were good to acceptable, ranging from α = 0.90 for contact intentions T1 to α = 0.72 for liking T2. Reliabilities for the indirect attitude measure, the IAT, were marginally acceptable (α = 0.61) for T1, but unsatisfactory for T2 (α = 0.59). Results concerning the IAT therefore must be interpreted cautiously, given the low reliability of this measure.

**TABLE 1:** Internal consistency reliabilities for the dependent measures used in the study.

Scale	Cronbach’s Alpha T1	Cronbach’s Alpha T2
RAS	0.85	0.87
NARS	0.82	0.82
MAS	0.76	0.72
Willingness to interact	0.81	0.84
Contact intentions	0.90	0.90
Liking	0.90	0.95
IDAQ	0.87	0.88
TA	0.88	0.87
IAT	0.61	0.59

#### 3.2.2 Qualitative Analyses of the Robot Stories

We furthermore subjected the stories which participants had produced about the everyday life of the robot NAO to a qualitative content analysis (Mayring and Fenzl, 2014). We explored whether the experimental manipulations might have led to differences in the produced written stories. To find out, we trained a research assistant in applying a coding scheme to analyze the following aspects of the robot stories: Length of the story, tone of the story (positive vs. negative, e.g., if the robot experienced negative emotions, if the robot was rejected, or if it was described negatively), whether the robot in the story had received a name (indicating anthropomorphizing), which activities the participants described, where the robot was located, whether or not the story included HRI, and whether or not participants had described emotions of the robot in their story (e.g., sadness, anger, joy, shame).

Results of the analyses showed that the word count ranged from 16 to 235 words (*M* = 97.38, *SD* = 50.70). The shortest story was because the particular participant had listed robot activities using bullet points instead of full sentences. As there was such a high variance in the overall length of the stories, we tested whether the word count significantly differed between conditions, which was not the case (*F*(2,52) = 0.69, *p* = 0.51). Furthermore, the texts were rated on whether the overall tone of the text was rather negative (−2) or rather positive (2), with an overall rating of *M* = 0.71 (*SD* = 0.99). This value implies that in general, the descriptions featured a neutral to positive tone. A rather negative story, for example, in parts read as follows: “Tobias, my owner, […], is not really interested in me anymore. […] As my possibilities are rather limited, the interest was gone again within a few minutes, and I got shut down and put back into the dark corner.” Again, we analyzed whether the tone varied between conditions, and did not find any significant differences (*F*(2,52) = 1.58, *p* = 0.22). However, even only at a descriptive level, it was interesting to note that the mean value for participants who were supposed to take the perspective of the robot was considerably lower (*M* = 0.39, *SD* = 1.09) compared to participants who were asked to suppress their stereotypes (*M* = 0.76, *SD* = 1.03) and compared to participants without further instruction (*M* = 0.95, *SD* = 0.83). In 16 cases, participants provided a first name for the robot in their story (29.09%), e.g., Cleanie, Joey, XYZ123, Robby, or Bob. In 39 stories, however, the robot remained unnamed (70.90%). 50 participants described a robot working (90.91%), e.g., at an office or at the university, two participants described the robot during leisure activities, e.g., playing soccer, in three stories both work, and leisure activities were described. (3.64%) However, only 23 stories described the robot at a workplace (41.81%), 20 participants described the robot being located at the owner’s home (36.36%). Three participants included several locations (5.45%), and nine participants did not include a location in their description (16.36%). 42 stories included an interaction between the robot and at least one human (76.36%), while 13 stories did not include any HRI (23.64%). In 22 of the produced stories, emotions of the robot were described (40%), e.g., “the robot is programmed to feel empathy”, “since then, it is feeling more comfortable in its environment”, or “Robi is sad”, 33 stories did not include any emotions of the robot (60%). As these data were categorical, we conducted chi-square tests to find out whether the stories significantly differed between conditions. We did not find significant differences between conditions on any of the variables, χ^2^(2) = 1.78, *p* = 0.47 for robot name, χ^2^(4) = 1.05, *p* = 0.94 for robot activity, χ^2^(8) = 5.17, *p* = 0.83 for robot location, χ^2^(2) = 1.31, *p* = 0.57 for HRI, and χ^2^(2) = 0.55, *p* = 0.79 for robot emotions. Therefore, there was no need to control for influences of the qualitative variables in the main analyses.

#### 3.2.3 Explicit Robot Attitudes

We analyzed participants’ pre-existing explicit robot attitudes, means and standard deviations are displayed in Table 2. Participants showed values slightly below the scale midpoint on RAS (*M* = 3.39, *SD* = 1.14) and NARS (*M* = 3.43, *SD* = 0.98), showing that their attitudes towards robots were not as negative as previously assumed, and that their robot anxiety was rather moderate. With regards to willingness to interact with robots, participants reported moderate interest (*M* = 4.76, *SD* = 1.27). Their actual contact intentions were lower (*M* = 3.66, *SD* = 1.76). However, with regards to robot liking, it could be shown that participants tended to dislike robots (*M* = 2.46, *SD* = 1.11). Their ascription of mind to robots was also moderate to low (*M* = 3.13, *SD* = 0.91), in light of a 7-point Likert scale.

**TABLE 2:** Mean values for explicit robot attitudes pre-intervention (T1).

Scale	Min	Max	*M*	*SD*
RAS	1.18	6.55	3.39	1.14
NARS	1.36	5.57	3.43	0.98
MAS	1.50	5.60	3.13	0.91
Willingness to interact	1.50	7.00	4.73	1.27
Contact intentions	1.00	7.00	3.66	1.76
Liking	1.00	5.86	2.46	1.11

#### 3.2.4 Intercorrelations Between Explicit and Implicit Attitude Measures

To gain an insight into the validity of the implicit robot attitude measure used in this study, we computed intercorrelations between the implicit and explicit attitude measures. Contrary to our expectations, there were very little significant correlations between implicit and explicit attitude measures. For the pre-intervention IAT we did not find any significant or trending correlations with measures of explicit attitudes, all *p*s > 0.10. The pre-intervention IAT only strongly and significantly correlated with the post-intervention IAT, *r* = 0.73, *p* < 0.0001. For the post-intervention IAT there were significant correlations with mind perception at T1, *r* = −0.36, *p* = 0.02, as well as with mind perception at T2, *r* = −0.33, *p* = 0.04. In this case a negative correlation is expected, as high values for the IAT score equal a higher preference for humans over robots, consequently people who preferred humans over robots to a stronger degree also ascribed less mind to robots. The lack of correlations with the attitude measures is rather surprising in the given context and needs to be considered when interpreting results regarding the IAT, as it seems that at least the post-intervention IAT rather served as a measure of implicit anthropomorphism than as a measure of implicit robot attitudes.

## 4 Main Analyses

### 4.1 Analyses Without Covariates

**FIGURE 1:**
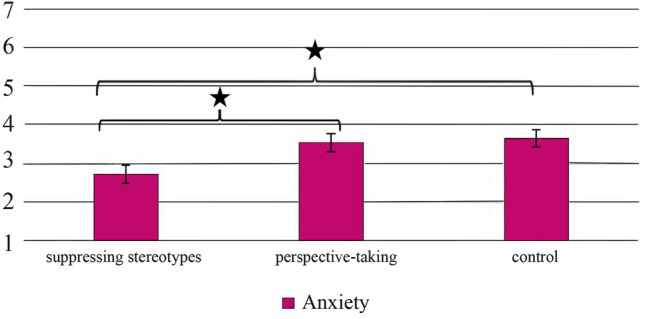
Mean values for robot anxiety across the experimental groups.

To test hypotheses 1a–2b, a mixed ANOVA was computed using the experimental condition (i.e., perspective-taking vs. suppressing stereotypes vs. control) as the independent variable, the factor *measurement time* (pre vs. post treatment, T1 and T2, respectively) as the repeated measures factor, and the dependent variables described above as dependent variables. Results showed that participants robot anxiety significantly differed between conditions, (*F*(2,52) = 4.03, *p* = 0.024, *η* ^2^ = 0.134), with the anxiety being significantly lower if participants wrote a story in which they were instructed to suppress their stereotypes about robots (*M* = 2.71, *SE* = 0.26), compared to when they took the perspective of the robot (*M* = 3.53, *SE* = 0.25, *t*(36) = 2.30, *p* = 0.08, which is a trend, *d* = 0.75) and compared to when there was no further instruction (*M* = 3.63, *SE* = 0.24, *t*(35) = 2.63, *p* = 0.03, *d* = 0.86), which is displayed in Figure 1. This result is contrary to Hypothesis 1a but supports Hypothesis 1b.

**FIGURE 2:**
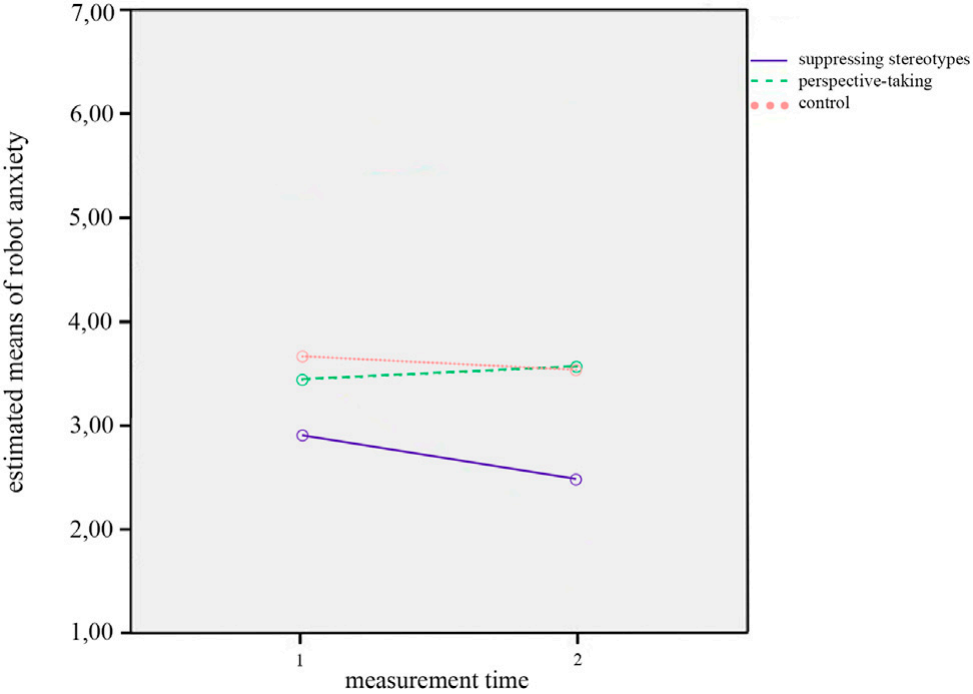
Interaction effect of measurement time by condition on anxiety.

Furthermore, there was a significant interaction effect of *measurement time* and *condition* on robot anxiety, *F*(2,52) = 3.062, *p* = 0.055, *η* ^2^ = 0.105. This is illustrated in Figure 2. This interaction effect indicates that the changes on robot anxiety between T1 and T2 differed across conditions. That is, while robot anxiety seemed to go down in the condition in which participants suppressed their stereotypes, as well as in the control condition in which participants did not receive further instructions, robot anxiety did not go down but rather increased when participants took the perspective of the robot when writing the story about its everyday life.

**FIGURE 3:**
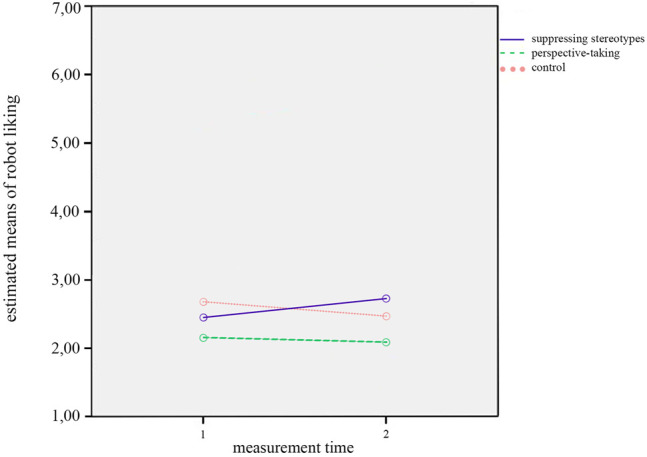
Interaction effect of measurement time by condition on robot liking.

Finally, a significant interaction effect between *measurement time* and *condition* on robot liking was obtained, *F*(2,52) = 3.33, *p* = 0.044, *η* ^2^ = 0.113. Figure 3 shows that the changes in robot liking between T1 and T2 varied across conditions: Neither in the control condition nor in the perspective-taking condition did writing a story about the life of a robot impact liking of robots. However, in the stereotype suppression condition liking of robots in general seemed to improve. On the contrary, writing a story about the everyday life of a robot seemed to decrease robot liking from T1 to T2 when participants were instructed to take the perspective of the robot, and when they did not receive any further instruction. There were no other significant effects on any of the dependent variables. The two interaction effects, as well as the fact that we did not find any significant effects of the factor *measurement time*, contradict Hypothesis 2a, which therefore cannot be confirmed. The interaction effects partially confirm Hypothesis 2b. However, as we did not find and significant main effects for the factor *measurement time*, there is no further support for Hypothesis 2b.

### 4.2 Analyses With Covariates

To make sure that the results found were in fact due to our experimental manipulation rather than being biased by covariates, we decided to conduct an additional mixed analysis of covariance (ANCOVA) and included the following covariates into the analysis: age, gender, robot experience, acceptance and usage of new technologies, and the individual proclivity to anthropomorphize. According to the principle of parsimony (Tabachnik and Fidell, 2013), covariates that do not significantly influence the dependent measures should be eliminated from the ANCOVA. Accordingly, age was removed as a covariate, as it did not significantly influence any of the dependent variables. Thus, the mixed ANCOVA included the experimental *condition* (perspective-taking vs. suppressing stereotypes vs. control) as the independent variable, the factor *measurement time* (pre vs. post treatment) as the repeated measures factor, the dependent variables described above, as well as the aforementioned remaining covariates.

Results showed a significant main effect of *measurement time* on robot liking (*F*(1,47) = 4.07, *p* = 0.049, *η* ^2^ = 0.08). However, when doing the post-hoc analyses, there was no statistically significant effect between any of the experimental groups, meaning that the effect only reached significance level due to alpha-error inflation in the first place. Furthermore, as in the previous analysis without covariates, we found a significant effect of the experimental condition on anxiety, *F*(2,47) = 3.75, *p* = 0.031, *η* ^2^ = 0.138. Participants who were instructed to write a story while suppressing their stereotypes reported significantly less anxiety (*M* = 2.81, *SE* = 0.24), compared to the control group (*M* = 3.70, *SE* = 0.22, *t*(35) = 2.71, *p* = 0.01, *d* = 0.89), which supports Hypothesis 1b.

**FIGURE 4:**
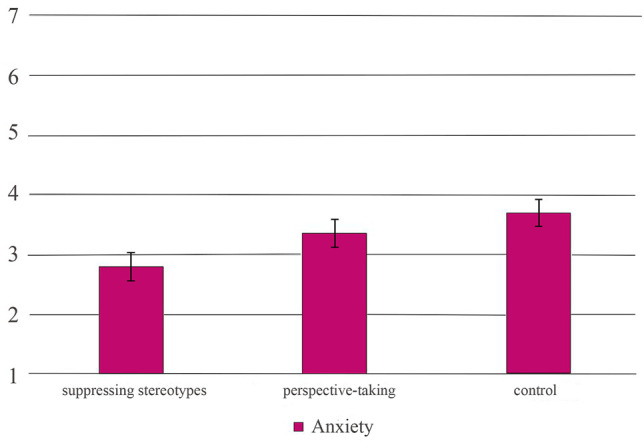
Mean values for robot anxiety as a function of experimental condition.

In contrast to the same analyses without the inclusion of covariates, there were no other significant differences between the groups, meaning that the previous difference between the group which suppressed their stereotypes and the group that was instructed to take the perspective of the robot lost significance when controlling for covariate influences. The results are displayed in Figure 4. We did not find any significant main effects on attitudes for the group that took the perspective of the robot. Therefore, Hypothesis 1b was not supported by the results.

**FIGURE 5:**
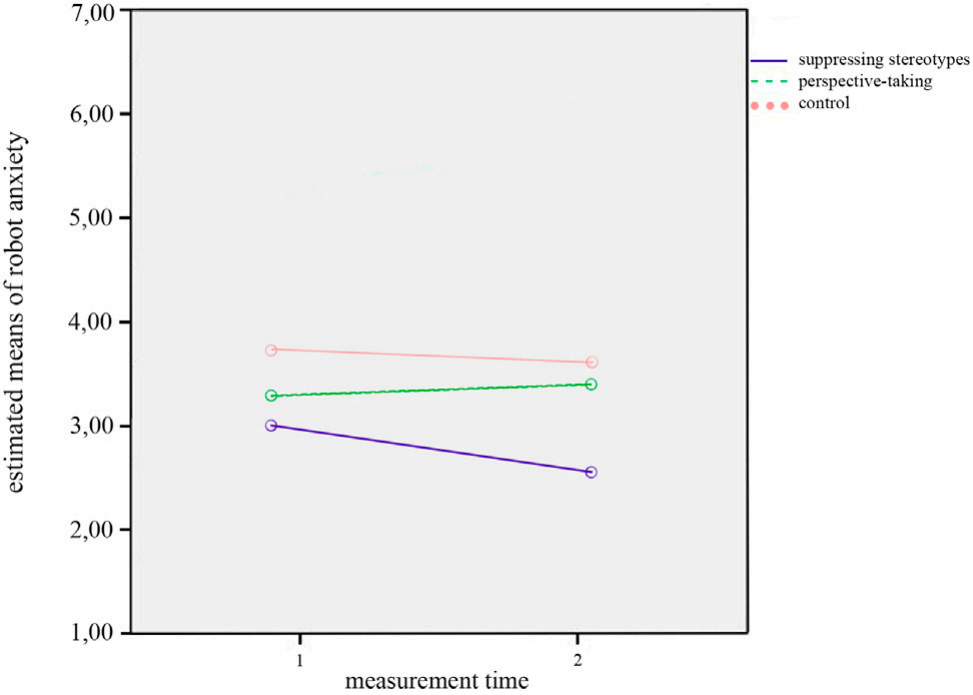
Interaction effect of measurement time by condition on anxiety.

However, we found a trend towards an interaction effect between *measurement time* and *condition* on anxiety, *F*(2,47) = 2.62, *p* = 0.084, *η* ^2^ = 0.10, which is shown in Figure 5. As for the interaction effects found in the analyses without covariates (see Section 4.1), this effect indicated that the changes on robot anxiety between T1 and T2 varied between conditions. Self-reported robot anxiety decreased when participants suppressed their stereotypes about robots, and likewise in the control condition (with a steeper decline in the stereotype suppression condition), while it slightly increased when participants took the perspective of the robot.

**FIGURE 6:**
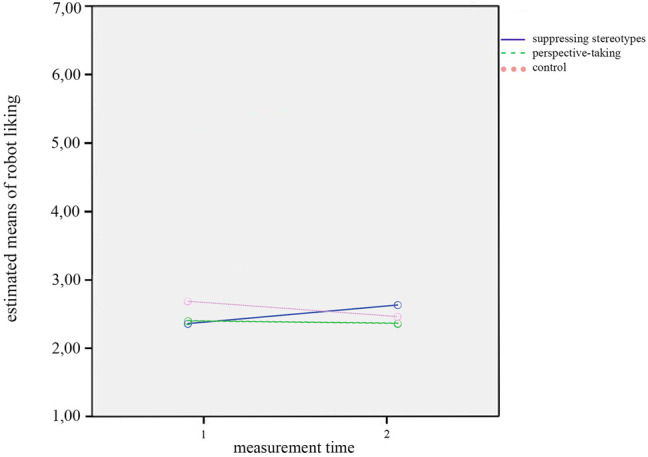
Interaction effect of measurement time by condition on robot liking.

Finally, we obtained a significant interaction effect between *measurement time* and *condition* on robot liking, *F*(2,47) = 4.36, *p* = 0.018, *η* ^2^ = 0.157. The interaction is displayed in Figure 6. The effect showed that the changes on robot liking between T1 and T2 varied between conditions. The interaction pattern for the results *with* covariates was similar to the one we found for robot liking in the analysis without covariates. However, in this case the graph indicating the stereotype suppression condition crossed both other condition graphs, showing that the group that suppressed their stereotypes benefited the most from the diversity training task. There was a rather steep incline in robot liking for the stereotype suppression condition, while the other two conditions showed a decline, which was especially pronounced for the control condition in this case.

There were no other significant effects (all *p*s > 0.10). The results of the interaction effects, as well as the fact that we did not find any significant effects of the factor *measurement time*, again did contradict Hypothesis 2a. With regards to Hypothesis 2b, the interaction effects partially supported Hypothesis 2b, but the nonsignificant results for the factor *measurement time* did not provide further support for this hypothesis.

## 5 Discussion

Stereotypes and prejudice are still a problem in society as a whole. Thus, these preconceived notions about what members of another group are like also impact judgments and behavior in the workplace context. In fact, previous research has shown that pre-existing stereotypes can hinder diverse teams at the workplace in unfolding their full potential (Ely et al., 2012). With robots becoming more and more prevalent, further “complicating” the picture, we were interested to test whether interventions used in the human-human context to manage diversity can potentially be beneficial for hybrid human-robot teams as well. Therefore, in the present study, we tested the effect of a diversity training task, more specifically a perspective-taking exercise vs. a stereotype suppression task, on implicit and explicit attitudes towards robots, to take a first step at finding out if diversity training might be an intervention that can change a-priori attitudes in mixed teams of humans and robots at the workplace. We predicted that the perspective-taking would positively influence robot attitudes and, therefore, show comparable beneficial effects as a diversity training task in the context of teams working together would. Contrary to H1 and H2a, perspective-taking did not improve robot attitudes. In fact, we observed that the stereotype suppression when reflecting on the everyday life of a robot had more beneficial effects on robot attitudes than the robot-perspective-taking alone. The present results further implied that participants did not particularly like taking the perspective of a robot. A first possible explanation for the negative effect of the perspective-taking task on robot attitudes can be derived from the literature on dehumanization. According to social psychological theorizing, different forms of dehumanization are proposed: Animalistic and mechanistic dehumanization. Animalistic dehumanization implies the denial of characteristics that are uniquely human, which leads to seeing the dehumanized group as animal-like. Mechanistic dehumanization is the denial of attributes that constitute human nature, seeing the dehumanized group as objects or as machine-like, Haslam, 2006). Even though it is most common to dehumanize other individuals or social groups, people also engage in self-dehumanization. (Bastian and Crimston, 2014). That is, they deny themselves essentially human characteristics. In that sense, it might be plausible that the instruction to take the perspective of a robot triggered mechanistic self-dehumanization (i.e., making participants feel more like a robot or an object, resulting in negative feelings towards themselves). Accordingly, having to put themselves in the shoes of a robot might have been an aversive experience for the participants, which led to more negative attitudes towards robots. Another aspect that goes hand in hand with this explanation is the possibility that taking the perspective of the robot made participants reflect upon general robot capabilities, e.g., not being able to feel, not being empathic etc. This, in combination with the fact that in this group they were not instructed to suppress their stereotypes and were not necessarily motivated to correct them, might have led to an increase in negative attitudes towards robots. Therefore, one interesting avenue for a follow-up study would be to combine both intervention approaches used in this study by instructing participants to write a story about the everyday life of a robot taking its perspective while suppressing their stereotypes about robots. This might revert the negative effects of the perspective-taking, especially if a mechanistic self-dehumanization is part of the reason for the backlash. If participants are instructed to suppress negative (e.g., mechanistic) stereotypes about robots while taking a robot’s perspective, they might not self-dehumanize and experience the intended positive effect on their robot attitudes. It might also be beneficial to make participants write down their concrete stereotypes about robots at the end of the study to gain insight into their content and the way they might have influenced their ratings.

Another potential explanation for the lack of positive effects of robot-perspective-taking on robot attitudes might be that participants did not feel comfortable putting themselves in the shoes of the robot, as people most possibly are not familiar with and/or must put more effort into building an idea of what a robot’s everyday life could be like. Data on participant’s prior robot experience revealed that more than two thirds of participants did have little to no robot experience, further supported this assumption. It could help to give participants a broad framework on how the everyday life of a robot might be like and provide them with a few keywords to help with generating the stories they were supposed to write. Trying to take the perspective of the robot might have put a considerable amount of cognitive load on the participant, due to his/her inexperience with a robot’s everyday life. It could be argued that reflecting on one’s own stereotypes of robots might also have put cognitive load on the participants, therefore introducing error variance into the experiment that needs to be controlled for. As we did not include a measure to assess participants’ cognitive load during the writing exercise, having more unpleasant experience during task fulfillment due to a higher amount of cognitive load cannot be ruled out as a possible explanation for the fact that perspective-taking led to an increase in negative robot attitudes. Therefore, based on this preliminary evidence, we recommend including a measure of cognitive load and/or task difficulty into following experiment using this writing exercise approach.

Additionally, the content of the stories the participants wrote might offer potential explanations for the lack of positive effect of the perspective-taking task on robot-related attitudes. The written stories showed a broad range of topics: A robot at home, a robot at the office, companion vs. service robot tasks etc. Even though we examined the variance of several factors between conditions, e.g., whether participants named the robot, whether HRI was described, or where the robot was located, and did not find any significant differences between conditions, it might be possible that some variables we did not think of might have influenced the results. Consequently, for follow-up studies, we recommend narrowing down the potential content of the stories by giving a more concrete instruction. This could be done, for instance, by asking participants to write down the everyday life of a robot that works as an assistant at home. Furthermore, it might be recommendable to not just show participants a picture of a robot, but to include information about the robot to narrow down what it might be capable of. By that, we at the same time make the experimental manipulation more comparable to a potential control group with human targets. As participants naturally have more experience with and exposure to the everyday life of a human, it can be assumed, that the range of content for such a group would be comparably narrower, if given no further instruction (e.g., a person eating breakfast, leaving for work etc.). In addition, it might be fruitful to include a direct measure on whether participants enjoyed the given task, including an open question format in which they can state reasons why they potentially disliked it. This open question format on why or why not participants liked or disliked the task and follow-up qualitative analyses could give insight into the reasons why participants potentially are averse to take the perspective of a robot.

### 5.1 Strengths, Limitations and Outlook

Our study realized a multi-method approach, including implicit as well as explicit measures. Moreover, we realized quantitative as well as qualitative analyses. This multi-method approach is a major strength of our study, potentially offering a very detailed insight into the effects of the utilized diversity training intervention on robot-related attitudes. However, the main hypotheses were not confirmed. There are several potential methodological reasons as to why that might be the case, which lead to further recommendations for future studies, making our results a fruitful basis for future research. One major limitation of the study concerns the participant number. The final sample only consisted of 55 people. The small sample size impacts the statistical power of the analyses, as two of the three groups even include data from less than 20 individuals. A post-hoc power analysis with G*Power showed that the statistical power to find small to medium effects for the analyses we carried out was 34% at most. To reach a power of 95%, a sample of 251 participants would have been necessary. As the results showed effects that were only trends, and some of the data were close to being trending, it might be possible that with a larger sample and more statistical power, these effects would reach statistical significance. This is especially important because analyses have shown that robot attitudes were not as negative as assumed to begin with, leading, to the possibility that some of the changes that may have occurred due to the intervention were rather small. For follow-up experiments we would recommend using an a-priori power analysis to predetermine the sample size necessary for the estimated effect size. On a related note, it might be beneficial to include measures of attitudinal ambivalence in future studies. Ambivalence is defined as “the simultaneous experience of both positive and negative feelings about one and the same attitude object” (Stapels and Eyssel, 2021, p.1). The fact that we found rather moderate ratings on robot anxiety as well as negative robot attitudes can possibly be explained by the fact that moderate ratings on robot attitudes have been shown to be due to attitudinal ambivalence. A moderate rating does not necessarily mean that attitudes are indeed moderate, but that people see negative as well as positive aspects of robots at the same time (Stapels and Eyssel, 2021).

Moreover, the duration of the study should be optimized for follow-up studies: Generally, diversity trainings are somewhat time-consuming and take place in the context of a workshop or the like, some of which last over several days. It is probable that a prolonged training with different tasks might have shown more, or stronger significant effects (in the expected as well as potentially in unexpected directions). It is recommendable for prospective research to include longer tasks, or vary task length as a factor, to test if this affects the results.

In addition, the limited reliability of the IAT as well as the inconsistent pattern of the correlations of the IATs with the explicit attitude measures must be considered, which is especially important as the fact that we did not find any significant results on the IAT might be, at least partly, due to reduced reliability values. The general problem of lower reliabilities of implicit attitude measures is common and has already been widely discussed in the literature (e.g., Nosek et al., 2005), so the fact that we found lower reliability values in the sample does not necessarily limit the value of the results. Nonetheless, it is notable that the already rather low reliability further dropped at T2. This might be due to signs of fatigue of the participants, as they had to execute the IAT twice (180 trials each time). Executing the IAT requires concentration and having to repeat the task might have been tiring for the participants, leading to vigilance problems. Unfortunately, as we were interested in changes in the data from T1 to T2, it was not possible to reduce the task number to one IAT for each participant. However, for future studies it might be beneficial to include instructions about the importance of staying vigilant and the problems associated with a less thorough task performance before the second IAT. The pattern of intercorrelations with regards to the implicit and explicit attitude measures implies that the post-intervention IAT can be considered as a measure of anthropomorphism instead of robot attitudes, while it remains unclear why the pre-intervention IAT did not correlate with any of the explicit measures while, at the same time, being highly correlated to the post-intervention IAT. One potential explanation for the lack of significant results may be the aforementioned low statistical power of the sample. A post-hoc power analysis revealed that the power to find small effects with regards to the correlations was just 11%, for medium effects it was 63 and 98% for large effects, which leads to the assumption that - given a bigger sample size - the pattern of correlations would have been different. Furthermore, it must be noted that there is ongoing debate on the validity of the IAT as a measure of attitudes (e.g., see Oswald et al., 2013; Rubinstein et al., 2018). However, in light of the existing results from meta-analyses which speak to the usefulness of this measure (e.g., Kurdi et al., 2019), we included it in the present experiment.

In addition, to rule out alternative explanations for potential results, for upcoming studies we plan to include entity (human vs. robot) as a factor and apply the same experimental tasks to human targets, or even vary ingroup vs. outgroup as a factor. That way it is directly comparable if the results differ between humans and robots, as our results imply.

Moreover, it might be interesting to test the replicability of the results on general robot attitudes if we used other robot types as stimuli. If unfamiliarity with a robot’s everyday life influenced the results, this effect might be even more pronounced when using different robot prototypes. The visual human likeness of the NAO robot we presented before the writing task in this study at least gave some hints on how the daily life and/or the tasks of the robot might be. If we used a totally different robot, e.g., a zoomorphic or a rather mechanomorphic robot, participants might use other clues to construct the robot’s daily life. The concept of humanity as a basis of their stories might not be as readily available as in the current study. Therefore, we recommend introducing robot type as a factor into follow-up experiments.

Additionally, many of the studies on diversity training in the human-human context directly aim at team performance and behavior. However, the current experiment featured the experimental reality of a laboratory experiment and, therefore, did not involve actual HRI. It also did not feature a real, physical robot, but a picture of a robot. Consequently, the insights from our experiment may not necessarily transfer directly to behavior in HRI and to the performance of hybrid human-robot teams and may differ when confronting participants with a real robot, especially as in-person interaction might already alter robot attitudes. Thus, it might be an interesting direction for follow-up research to find out whether the results we found on the measures of robot attitudes transfer to actual behavior. On the one hand, it would be possible to capture behavioral intentions more directly than we did with the used measures of general contact intentions and willingness to interact, i.e., by announcing that participants would have the opportunity to interact with a robot after the experiment and ask them whether they would like to take the opportunity. On the other hand, an actual HRI could be included into the experiment and verbal as well as non-verbal interaction behavior by the participant could be measured. Including actual behavioral measures would also allow to test a team’s productivity pre- and post-intervention to determine whether a diversity training can indeed influence team productivity. Furthermore, in an experiment utilizing real HRI it would be possible to measure perceived as well as objective cooperation quality. It might also be interesting to conduct a field experiment in a workplace setting in which robots are supposed to be introduced, which will probably occur with a higher frequency in the future. Another potential route for future research could be to use video material of the robots instead of relying on images alone. That way, the participants could get more information about the potential capabilities of the robot (e.g., by seeing it move and talk). Furthermore, watching a video of a robot that indeed moves and reacts could increase the external validity of the design compared to solely using picture material. On a related note, our sample did have very little to no robot experience. We chose such a naïve sample as we emanated that it makes sense for a diversity training in the human-robot context to be conducted when robots are introduced into a workplace. Participants of the training would therefore be comparably unfamiliar with robots. However, it is important to consider the possibility that results may differ for situations in which robots have already been part of the workplace and the diversity training intervention is used to improve the hybrid human-robot team, as people in such situations already have more experience with robots, which may, for example, lead to stronger robot-related attitudes. Furthermore, it might be helpful to provide the participants with more context, i.e., task scenarios, in advance. As, according to task technology fit theory (e.g., Furneaux, 2012), system use and performance benefits are related to the fit of the system to the task, the specific setting might influence results according to whether or not participants found the robot suitable for the area of use and the tasks they described.

One final remark refers less to the study we conducted but more to the general usage of diversity training in the HRI context. We have previously mentioned the aspect of mechanistic dehumanization, and using diversity training interventions in the context of humans and robots may lead to the impression that human outgroups are equalized with robots. This may feel discriminatory or even lead to said dehumanization. To make sure that this impression does not form, and that there is no discrimination based on this, it probably is recommendable to give diversity trainings in the context of humans and robots another, new name that does not carry such connotations, e.g., human-robot team training or a comparable term.

## 6 Conclusion

To sum up, the approach to diversity training we utilized in this study, namely, the enlightenment approach (Dovidio et al., 2004), yielded surprising results: It did not improve robot-related attitudes. On the contrary, it seemed stereotype suppression was more successful in improving robot-related attitudes. Apart from potential methodological reasons, results imply that people dislike taking a robot’s perspective. It is necessary to take this potential aversion into account when designing or utilizing interventions to improve robot attitudes and/or prepare people for HRI and human-robot cooperation in hybrid human-robot teams. More specifically, the preliminary results suggest refraining from using exercises that use perspective-taking when the goal is to improve attitudes in hybrid human-robot teams. Instead, it might prove beneficial to merely provide facts and information about robots before introducing hybrid teams. That way, potential human co-workers’ awareness concerning their own preconceived notions about robots and other innovative technologies can be raised. Ultimately, such information politics may represent a simple and easy way to facilitate human-robot collaboration that is deemed not only subjectively positive, but also objectively productive.

## Data Availability

The original contributions presented in the study are included in the article/Supplementary Material, further inquiries can be directed to the corresponding author.
