# Supplementary figures and images for: Diversity Training With Robots: Perspective-Taking Backfires, While Sterotype-Suppression Decreases Negative Attitudes Towards Robots
Source: Front Robot AI. 2022 Mar 9;9:728923. doi: 10.3389/frobt.2022.728923 (PMC8959608; doi:10.3389/frobt.2022.728923)

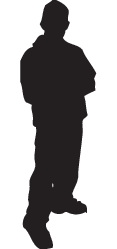

Supplement: Supplementary file 1 [file Presentation1.ZIP › m1.bmp]

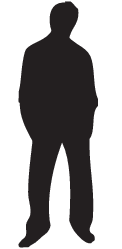

Supplement: Supplementary file 1 [file Presentation1.ZIP › m10.bmp]

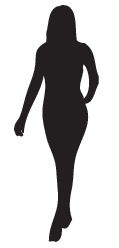

Supplement: Supplementary file 1 [file Presentation1.ZIP › m2.bmp]

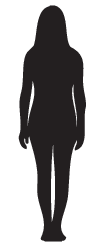

Supplement: Supplementary file 1 [file Presentation1.ZIP › m3.bmp]

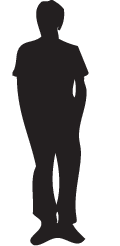

Supplement: Supplementary file 1 [file Presentation1.ZIP › m4.bmp]

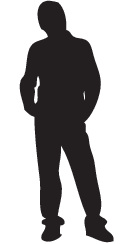

Supplement: Supplementary file 1 [file Presentation1.ZIP › m5.bmp]

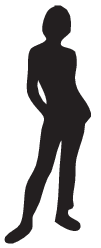

Supplement: Supplementary file 1 [file Presentation1.ZIP › m6.bmp]

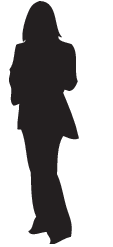

Supplement: Supplementary file 1 [file Presentation1.ZIP › m7.bmp]

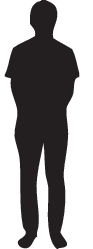

Supplement: Supplementary file 1 [file Presentation1.ZIP › m8.bmp]

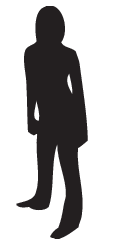

Supplement: Supplementary file 1 [file Presentation1.ZIP › m9.bmp]

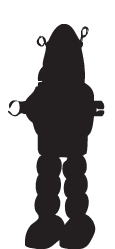

Supplement: Supplementary file 1 [file Presentation1.ZIP › r1.bmp]

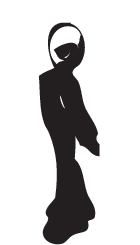

Supplement: Supplementary file 1 [file Presentation1.ZIP › r2.bmp]

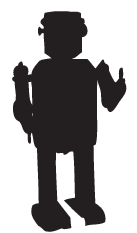

Supplement: Supplementary file 1 [file Presentation1.ZIP › r3.bmp]

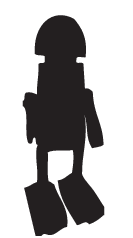

Supplement: Supplementary file 1 [file Presentation1.ZIP › r4.bmp]

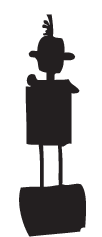

Supplement: Supplementary file 1 [file Presentation1.ZIP › r5.bmp]

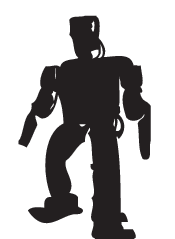

Supplement: Supplementary file 1 [file Presentation1.ZIP › r6.bmp]

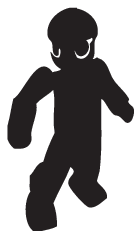

Supplement: Supplementary file 1 [file Presentation1.ZIP › r7.bmp]

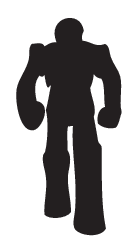

Supplement: Supplementary file 1 [file Presentation1.ZIP › r8.bmp]
